# The therapeutic potential of *Vanilla planifolia*: how its phytochemicals combat diabetes?

**DOI:** 10.3389/fphar.2026.1752315

**Published:** 2026-04-13

**Authors:** Bee Ling Tan, Fatimah Zulkifli, Mohd Esa Norhaizan

**Affiliations:** 1 Department of Diagnostic and Allied Health Science, Faculty of Health and Life Sciences, Management and Science University (MSU), Shah Alam, Selangor, Malaysia; 2 Department of Nutrition, Faculty of Medicine and Health Sciences, Universiti Putra Malaysia, Serdang, Selangor, Malaysia

**Keywords:** antioxidant, bioactive compound, diabetes, inflammation, oxidative damage, oxidative stress, phytochemical, polyphenol

## Abstract

Oxidative stress is defined as an imbalance between pro-oxidant and antioxidant species, resulting in molecular and cellular damage. It is recognized as a key pathogenic mechanism contributing to the initiation and progression of diabetes. Emerging research suggests that low-grade chronic systemic inflammation plays a crucial role in the development and progression of various chronic diseases including diabetes. *Vanilla planifolia*, a widely valued spice known for its distinctive sweet aroma and flavor, is primarily obtained from its beans and extensively used worldwide. *Vanilla planifolia* is a tropical plant valued not only for its aromatic pods but also for its diverse pharmacological properties. Vanillin is the primary bioactive compound in vanilla, contributing to its distinct flavor and aroma, alongside other phenolic compounds with antioxidant properties. Vanillin regulates antioxidant and anti-inflammatory pathways by enhancing insulin sensitivity, reducing inflammation by downregulating NF-κB, and upregulating AMPK and GLUT4 translocation. This review synthesizes current findings on the therapeutic prospects of *Vanilla planifolia*, emphasizing its relevance as a natural source of bioactive compounds with significant implications for diabetes.

## 1 Introduction

Diabetes mellitus continues to be a growing global burden, remaining one of the greatest public health challenges of the twenty-first century. With the increasing global prevalence of both Type 1 diabetes mellitus (T1DM) and Type 2 diabetes mellitus (T2DM), effective approaches to prevention, management, and complication prevention are needed (Jamil et al., 2024). Although both T1DM and T2DM are characterized by chronic hyperglycaemia, T1DM is driven by autoimmune destruction of insulin-producing β-cells (Syed, 2022), and T2DM is driven by a combination of insulin resistance and β-cell dysfunction (Cohrs et al., 2020). Regardless of the kind, blood sugar levels have significant effects on managing blood glucose levels and preventing chronic issues, especially when it comes to their lifestyle.

Functional foods and plant-derived bioactives, particularly phytochemicals, are the result of the search for a compound or compounds with a safe and non-invasive therapeutic approach. These compounds have anti-inflammatory properties, regulate blood glucose, and may also play a role in glycaemic control and metabolic process modulation (Golovinskaia and Wang, 2023). However, scientific interest in this area has grown, particularly around *Vanilla planifolia*, a tropical orchid most notably recognized for its fragrant vanilla beans but which has gained popularity for having a plethora of phytochemical components and potential benefits to treat prevailing metabolic diseases such as diabetes (Olędzka and Czerwińska, 2023).

Traditionally recognized for its flavour and fragrance, *Vanilla planifolia* is rich in bioactive compounds, including vanillin, vanillic acid, polyphenols, and phenolic acids, all of which display antioxidant, anti-inflammatory, and hypoglycaemic properties (Kafali et al., 2024). These compounds are thought to alter essential mechanisms involved in diabetes pathophysiology via mitigation of oxidative stress, increased insulin sensitivity and attenuation of inflammatory signalling that mediate glucose and lipid homeostasis (Sibony et al., 2024).

Since diabetes is a group of syndromes caused by multifactorial and pathophysiological causes (Rachfal et al., 2021), recent studies have shown that *Vanilla planifolia* can serve as a functional food ingredient with beneficial effects on various aspects of modulation of diabetes pathology, yet not limited to one type of diabetes (Olędzka and Czerwińska, 2023). The most significant phytochemicals in vanilla (vanillin and vanillic acid) have been reported to modulate glucose uptake, inflammatory markers, and oxidative homeostasis pathways known to play a role in T1DM and T2DM. Furthermore, these compounds seem to promote beta cell survival and enhance insulin signalling, as well as to inhibit chronic inflammation, making them particularly interesting in the broader context of diabetes mellitus (Ji et al., 2020). While previous studies have primarily focused on the general antioxidant and anti-inflammatory properties of *Vanilla planifolia* or its isolated compounds, this manuscript systematically elucidating how its diverse phytochemicals act through integrated molecular pathways to combat diabetes, including insulin receptor modulation, glucose transporter regulation, and inflammatory pathway suppression, revealing synergistic interactions that underpin the therapeutic potential of *Vanilla planifolia*. The safety and toxicity of vanillin was also highlighted in this study.

## 2 Oxidative stress

The detrimental effects of free reactive nitrogen species (RNS) and reactive oxygen species (ROS) can lead to significant biological damage, manifesting as nitrosative and oxidative stress, respectively (Tan and Norhaizan, 2024a). ROS are naturally produced as by-products of aerobic metabolism; however, under stress conditions, their elevated levels pose serious health risks (Tan et al., 2024). The mitochondrion is the primary cellular organelle responsible for ROS generation (Tan et al., 2018a), producing adenosine triphosphate (ATP) through oxidative phosphorylation. During this process, incomplete reduction of oxygen via one- or two-electron transfers instead of the typical four-electron reduction results in the formation of hydrogen peroxide (H_2_O_2_) or superoxide anion (O_2_∙^-^), which can subsequently give rise to other ROS.

Major RNS include nitric oxide (NO) and peroxynitrite (ONOO^−^) (Tan and Norhaizan, 2024a). Excess NO can react to form nitrogen dioxide radicals, while elevated NO concentrations may lead to the generation of dinitrogen trioxide (N_2_O_3_), promoting nitrosation reactions. Oxygen-derived free radicals such as alkyl peroxyl radical (?OOCR), hydroxyl radical (OH∙^-^), and superoxide anion (O_2_∙^-^) are potent initiators of lipid peroxidation, a process strongly implicated in the pathogenesis of various diseases (Tan et al., 2024). Once initiated, lipid peroxidation propagates through chain reactions until termination products are formed. These end products, including F_2_-isoprostanes, 4-hydroxy-2-nonenal (4-HNE), and malondialdehyde (MDA), accumulate within biological systems (Poljšak et al., 2025).

DNA bases are highly susceptible to oxidative damage by ROS, with 8-hydroxy-2-deoxyguanosine being the most commonly detected oxidation product (Hong et al., 2024). Such modifications can result in mutations and deletions in both nuclear and mitochondrial DNA. Mitochondrial DNA is particularly vulnerable due to its close proximity to ROS and its limited repair mechanisms compared to nuclear DNA. Oxidative alterations also affect structural and enzymatic proteins, potentially leading to significant physiological consequences. Furthermore, redox-mediated changes in transcription factors can modulate their DNA-binding activity, thereby influencing gene expression (Tan et al., 2018b).

## 3 Diabetes mellitus

### 3.1 Overview of diabetes mellitus

Diabetes had significant global implications, contributing to an estimated 1.5 million deaths in 2019, nearly half of whom were under 70 years of age (WHO, 2024). It is one of the most common global health problems faced today, with rapidly increasing incidence. If trends continue, this figure is estimated to rise to 643 million by 2030 and to 783 million by 2045 (International Diabetes Federation, 2021). Statistics from 2021 indicate the Western Pacific Region, comprising Malaysia, China, Japan, and Australia, has the highest incidence of problems, with 206 million diagnosed cases. Research indicates that the number of diabetics worldwide will rise from 171 million in 2021 to 260 million in 2045 because of ageing populations alongside urban growth and increased obesity levels (International Diabetes Federation, 2021).

The metabolic disease of diabetes mellitus produces ongoing high blood glucose that arises from insulin deficiency or insulin resistance in target tissues. The hormone insulin, which the pancreas produces, functions as a key mechanism to decrease blood glucose because it drives cell glucose absorption and suppresses liver glucose creation (Zheng et al., 2020).

Insulin resistance functions as a primary factor that promotes type 2 diabetes by diminishing tissue cells’ sensitivity to insulin in muscle, liver and adipose tissues. This impaired responsiveness disrupts normal glucose homeostasis, resulting in hyperglycemia. Over time, insulin resistance also contributes to a cascade of metabolic complications, including oxidative stress imbalance, chronic low-grade inflammation, cardiovascular dysfunction, and the onset of metabolic syndrome (Wolosowicz et al., 2022). In healthy individuals, insulin tightly regulates glucose uptake and production; However, in insulin-resistant states, these processes are dysregulated, leading to the characteristic high blood sugar levels of diabetes.

Malaysia is one of the countries with the highest prevalence of diabetes in the Western Pacific Region. According to the NHMS, 2023, diabetes affects 15.6% (or 1 in 6) of people in Malaysia, and concerningly, 2 in 5 of them are unaware of their illness. Although the frequency of diabetes rises with age, it may also afflict young adults (84% of people with diabetes between the ages of 18 and 29 are unaware that they have the disease). Given that diabetes continues to rank among the nation’s top causes of mortality, this lack of awareness is alarming (NHMS, 2023). This growing trend presents major healthcare and economic challenges, and Malaysia is anticipated to attain the status of an aged nation by 2030, whereby more than 14% of the population will consist of individuals aged 65 years and above, which will place additional strain on the burden of non-communicable diseases (NCDs), including diabetes (World Bank, 2020). In Malaysia, the economic burden of diabetes management is high, with annual healthcare costs amounting to over RM 9.65 billion (∼USD 2.1 billion), inclusive of treatment, hospitalization, and productivity losses (WHO, 2022).

Obesity and a sedentary lifestyle are major risk factors for type 2 diabetes, which accounts for approximately 80% of all diabetes cases (Laakso and Fernandes Silva, 2022). In addition, genetic predisposition plays a critical role in the development of diabetes by influencing glucose metabolism and insulin sensitivity (Ali et al., 2022). Recent developments in nutrigenomics, which show how nutrition influences gene expression, have created new opportunities for diabetes treatment and prevention through individualized dietary strategies (Felisbino et al., 2021).

### 3.2 Complications of diabetes

Diabetes mellitus is a chronic metabolic disorder that, when not well controlled, may lead to a spectrum of acute and chronic complications. These are due to long-standing elevated blood sugar (hyperglycemia), which damages various organs and systems in the body (Goyal et al., 2023). The two principal types of diabetes, Type 1 and Type 2, have many common complications, whereas Type 2 is primarily associated with insulin resistance and comorbidities such as obesity and hypertension (Sinha and Haque, 2022). DM complications can be broadly categorized into microvascular, macrovascular & neurological types, each contributing to the disease’s complexity and the challenges involved in its management (Paul et al., 2025).

Microvascular complications of diabetes are not particularly prevalent in regard to an increase in complications, however, these complications are particularly concerning because they involve damage to the small blood vessels (capillaries) of the various organs. These later complications are often a direct result of prolonged hyperglycaemia. They can significantly affect the body, especially the eyes, kidneys and nerves. Diabetic retinopathy, for instance, is among the leading causes of blindness and visual impairment among adults (Horton and Barrett, 2020). It is caused by damage to the blood vessels in the retina, the light-sensitive tissue at the back of the eye. Over time, the high blood glucose levels cause the damaged blood vessels to leak fluid, which can cause swelling, along with the growth of fragile new vessels that can bleed or block vision (Gale et al., 2021). Diabetic retinopathy occurs in stages, starting with non-proliferative retinopathy, which rarely displays symptoms, and may advance into proliferative retinopathy that can lead to significant loss of vision (Ren et al., 2022). Early diabetic retinopathy can be prevented by regular eye examinations, good blood glucose control, and blood pressure management (Gale et al., 2021).

Beyond vision problems, diabetes can also lead to one condition called diabetic nephropathy, in which chronically high blood sugar levels damage the tiny blood vessels found in the kidneys, limiting their ability to filter waste and excess fluids out of the bloodstream (Samsu, 2021). That damage can eventually lead to kidney dysfunction and, ultimately, failure. Diabetic nephropathy is among the most common causes of end-stage renal disease (ESRD), which can lead to dialysis or kidney transplantation. An early sign of diabetic nephropathy is proteinuria, the presence of protein in the urine, which is an early sign of kidney damage (Chen et al., 2022). Strict glycaemic and blood pressure control and routine monitoring of renal function are required to prevent or slow the progression of diabetic nephropathy (Samsu, 2021).

Macrovascular events can be accurately predicted by microvascular problems. For example, a higher risk of cardiovascular events is linked to diabetic retinopathy and nephropathy (Avogaro and Fadini, 2019). Patients with diabetes who have microvascular illness are at an increased risk of serious adverse cardiovascular events, especially ischaemic stroke (Olesen et al., 2022). Chronic hyperglycaemia leads to the thickening and stiffening of blood vessels (atherosclerosis), which raises the risk of heart attacks, stroke and heart failure. Diabetes is one of the most potent risk factors for coronary artery disease (CAD) that can result in heart attacks (Mastrogiacomo et al., 2022). Peripheral artery disease (PAD), which restricts blood flow to the legs and feet, is another condition that people with diabetes are more likely to develop. Patients undergoing lower extremity revascularisation are also more likely to experience major adverse cardiovascular events and major limb amputation, though not mortality (Edjimbi et al., 2025).

## 4 Role of oxidative stress in diabetes

Oxidative stress is recognized as a major contributing factor in the pathogenesis of diabetes mellitus. Multiple risk factors including aging, poor dietary habits, and obesity, create an oxidative environment that disrupts insulin sensitivity by promoting insulin resistance or impairing glucose tolerance. The underlying mechanisms are multifaceted, involving numerous cell signaling pathways. Hyperglycemia, a hallmark of diabetes, further exacerbates oxidative stress and accelerates disease progression. Both macrovascular and microvascular complications, which significantly contribute to the morbidity and mortality of diabetic patients, are closely linked to oxidative stress (Tan et al., 2025).

In type 2 diabetes, molecular and cellular dysfunctions are particularly evident in pancreatic β cells. Reactive oxygen and nitrogen species (ROS and RNS), including hydrogen peroxide (H_2_O_2_), superoxide anion (O_2_∙^-^), nitric oxide (NO), peroxynitrite (ONOO^−^), and hydroxyl radical (OH∙), play critical roles in disrupting physiological and metabolic processes. Mitochondrial dysfunction impairs ATP production, which compromises β-cell glucose stimulated insulin secretion (GSIS), NADPH complex activity, and calcium signaling essential for neurotransmission (Qin et al., 2025).

Insulin resistance is central to the metabolic disturbances associated with obesity. It reflects a diminished cellular response to insulin in key tissues such as skeletal muscle, adipose tissue, liver, and brain (Szablewski, 2024). This impairment reduces glucose uptake and increases hepatic glucose output, leading to elevated plasma glucose levels. The resulting disruption in glucose homeostasis places increased demand on pancreatic β cells to secrete insulin, a compensatory mechanism that may temporarily normalize blood glucose levels during early or prediabetic stages. However, persistent insulin resistance and chronic exposure of β cells to elevated glucose and lipid levels can lead to β-cell dysfunction and eventual onset of overt diabetes (Diane et al., 2024).

Pancreatic islets, highly vascularized and composed of five distinct cell types, α, β, δ, γ (ghrelin), and pancreatic polypeptide (PP) cells, regulate nutrient levels in the bloodstream. These islets receive blood supply from the splenic and pancreaticoduodenal arteries and coordinate nutrient sensing and hormone secretion. During nutrient deprivation, α and β cells modulate glucagon and insulin release, respectively. The β cell response to glucose is tightly regulated by intracellular and extracellular ROS and RNS levels (Tan et al., 2018a). Increased glycolytic flux enhances ATP production and oxidative phosphorylation, leading to superoxide generation from the electron transport chain. An adaptive response via the pentose phosphate pathway diverts excess glucose to pentose synthesis, but this may also activate NADPH oxidase (NOX), further increasing superoxide production. Additionally, elevated glucose levels can enhance ROS through mechanisms such as advanced glycation end product (AGE) formation and glucose autoxidation (Tan et al., 2018b).

Upon insulin release, its anabolic effects are mediated through the transmembrane insulin receptor on target tissues. Insulin binding triggers receptor autophosphorylation, recruitment of insulin receptor substrate (IRS) proteins, and activation of downstream signaling pathways including phosphatidylinositol-3-kinase (PI3K) and protein kinase B (Akt). Akt plays a pivotal role in translocating glucose transporter type 4 (GLUT-4) to the plasma membrane, facilitating glucose uptake in insulin-responsive tissues (Chantarasakha et al., 2025).

Studies have demonstrated increased protein nitrosylation and carbonylation in insulin-resistant and obese phenotypes, particularly within insulin-sensitive tissues (Tan and Norhaizan, 2024b). These modifications may downregulate insulin receptor expression. Chronic hyperinsulinemia and hyperglycemia, coupled with elevated ROS and RNS levels, are thought to alter insulin receptor gene expression via transcription factors such as high mobility group AT-hook 1 (HMGA-1). They may also enhance receptor desensitization, a process typically governed by negative feedback mechanisms (Tan et al., 2018a). Collectively, the progression of diabetes mellitus is driven by β-cell dysfunction and insulin resistance, both of which are closely associated with obesity.

## 5 Pathogenesis of type 2diabetes mellitus

Type 2 diabetes mellitus (T2DM) is a heterogenic disease due to interrelated factors resulting predominantly from insulin resistance and failure of beta cells (Rodríguez et al., 2021). In contrast to T1DM, where the fundamental problem is the absence of insulin production, in T2DM there is impaired insulin action on cells. This causes the concentration of glucose in the blood to rise, leading the pancreas to secrete more insulin in a bid to counteract the condition. However, the beta cells exhaust over time and can no longer secrete sufficient insulin, worsening the hyperglycemia (Esser et al., 2020).

Genetic and environmental factors both play a role in the pathogenesis of T2DM (Singh et al., 2024). There is a strong association between the growing rates of T2DM and the increasing prevalence of obesity, sedentary lifestyle and a poor-quality diet, particularly one high in refined carbohydrates and sugars. These factors lead to a decreased response of muscle, liver, and adipose tissues, known as insulin resistance (Chater et al., 2020).

In response to persistent insulin resistance, beta cells in the pancreas initially compensate by increasing insulin output. But this compensatory response ultimately breaks down as the pancreatic beta cells become surpassed by the continual need for insulin. Due to secretory exhaustion of the remaining beta cells, insulin secretion gradually declines, which worsens hyperglycemia (Castell et al., 2020). Visceral fat (located around the abdominal organs) further participates in the inflammatory mechanisms that interfere with insulin signalling (Szukiewicz, 2023). Adipokines (cytokines of the immune system that are produced by fat cells) inhibit pathways related to the insulin receptor, thereby enhancing insulin resistance and further corrupting glucose metabolism (Dongre, 2021).

Mitochondrial dysfunction contributes to the pathogenesis of T2DM, along with insulin resistance and beta cell dysfunction. Impaired energy-producing function of mitochondria stimulates fatty acid release and promotes oxidative stress, which inhibits the function of insulin and causes insulin resistance in T2DM (Huynh et al., 2023).

## 6 Mechanisms leading to insulin resistance

Type 2 diabetes mellitus (T2DM) predominantly features insulin resistance while this condition rarely affects Type 1 diabetes mellitus (T1DM) disease development. The glucose metabolic disruption in both diabetes types shows that insulin resistance plays a central role in T2DM diagnosis.

### 6.1 Insulin resistance in type 2 diabetes mellitus

T2DM causes insulin resistance that develops when cells from the liver and skeletal muscle and adipose tissue lose their normal insulin responsiveness and consequently increase blood glucose levels. Type 2 diabetes advances because of resistance which functions as a primary factor in disease development (Galicia-Garcia et al., 2020). Different factors work together to produce insulin resistance as a key feature of T2DM. The first step of insulin action happens when the hormone binds to cell surface receptor which triggers a succession of cellular processes inside the cell. The insulin signaling cascade starts when insulin receptor substrates (IRS) become activated particularly IRS-1 and IRS-2 before activating phosphoinositide 3-kinase (PI3K). Through PI3K activity GLUT4 relocates to cell membranes thus promoting glucose absorption.

The molecules that regulate IRS proteins in insulin resistance lose their capability for tyrosine phosphorylation but gain increased exposure to serine/threonine phosphorylation. Downstream signalling pathway dysfunction because of this disruption leads to poor glucose uptake and development of hyperglycemia (Pei et al., 2022). The path to insulin resistance depends heavily on the lipotoxicity pathway. Consuming high-fat diets causes fatty acids to build up in non-adipose tissues particularly liver and skeletal muscle as these areas disrupt insulin signalling pathways. Lipid intermediates including diacylglycerols (DAGs) and ceramides can activate protein kinase C (PKC) which leads to serine residue phosphorylation in IRS proteins and worsens insulin signalling (Lair et al., 2020). The excessive build-up of lipid intermediates worsens due to mitochondrial dysfunction that results in overwhelming fatty acid levels beyond mitochondrial oxidative capacity producing reactive oxygen species that lead to oxidative stress and ultimately insulin resistance (Frangos et al., 2021).

Insulin resistance develops significantly because of persistent low-level inflammation which becomes most pronounced in visceral fat tissue. Obesity triggers abdominal fat cells to release inflammatory cytokines that degrade insulin signaling pathways. Through cytokine activation serine kinases start a phosphorylation process of IRS proteins at serine residues leading to protein dysfunction which promotes insulin resistance (Gasmi et al., 2021).

## 7 Vanilla planifolia

The tropical climbing plant *Vanilla planifolia* known as the vanilla orchid grows naturally in Mexico together with Central America. The species *Vanilla planifolia* represents the most commercially grown member of the vanilla genus and functions as the basic natural flavor ingredient which appears in both food along with cosmetic and pharmaceutical products (Yusuf et al., 2023). The bioactive substance vanillin exists among various components of *Vanilla planifolia* which demonstrate anti-inflammatory and antioxidant properties. *Vanilla planifolia* contains important compounds which manage inflammation and oxidative stress that serve as key factors in metabolic disease development (Arya et al., 2021).

After harvesting, the vanilla plant produces green seed pods that growers call vanilla beans, which require extensive curing procedures. Pure vanilla pods include glucovanillin, which functions as the pod’s glycosylated non-aromatic starting material before converting into the main aromatic vanilla compound known as vanillin. The iodated pod cells contain the β-glucosidase enzyme within their cytoplasm, which activates during curing to split glucovanillin into vanillin through hydrolysis. After compartmentalising precursor substances from enzymatic components, cellular membranes require breakdown through curing to enable biochemical interactions which produce aromatic compounds (Delgado et al., 2021).

**FIGURE 1:**
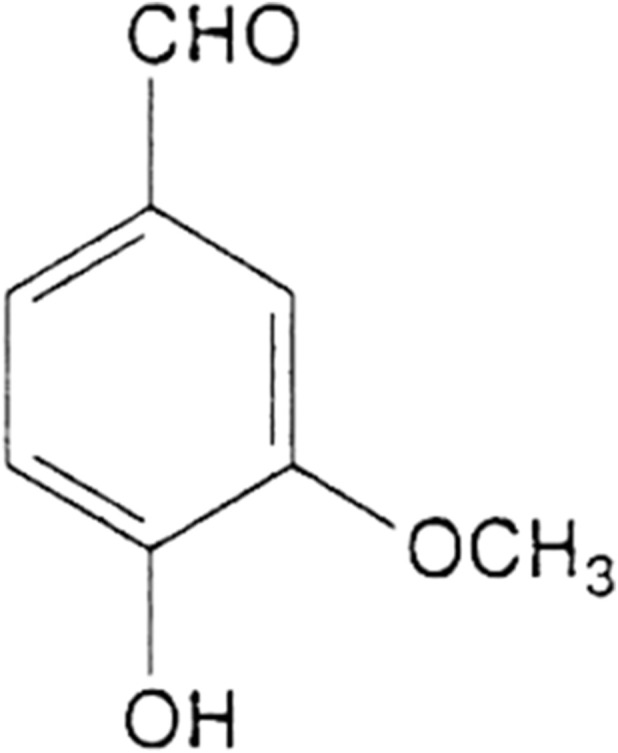
Molecular structure of vanillin.

The components of cured vanilla beans comprise 2% vanillin and multiple complex elements, including vanillic acid and oxalates, together with oleoresins, sugars and aldehydes (Liaqat et al., 2023). The phytochemical makeup of vanilla includes components which generate distinctive sensory experiences as well as therapeutic benefits. The pharmacological research on cured vanilla beans shows extensive investigation of vanillin and ferulic acid because these compounds demonstrate antioxidant capacities and anti-inflammatory properties with additional benefits for diabetes management, cardiovascular health and neuroprotective functions (Olatunde et al., 2022). Primary sources for obtaining the chemical vanillin (4-hydroxy-3-methoxybenzaldehyde) exist either in *the Vanilla planifolia* species or the hybrid variety *Vanilla tahitensis*. Ferulic acid (4-hydroxy-3-methoxycinnamic acid) represents a major wall plant cell component which co-occurs with vanillin in extract processes of plant materials. Figure 1 shows the molecular structure of vanillin.

The human diet contains polyphenolic compounds including vanillin and ferulic acid which consumers obtain through eating plant materials or because of curcumin breakdown (Zhupanova and Ziyatdinova, 2021). Scientists have established curcumin breaks down to vanillin and ferulic acid metabolites during the cooking process and after digestion resulting in urine concentrations exceeding curcumin by 1000-fold. Research suggests that curcumin breakdown produces metabolites that transmit multiple health benefits traditionally linked with curcumin such as antioxidant defense and reduction of inflammation and insulin sensitivity (Bozkurt et al., 2022). Both vanillin and ferulic acid show therapeutic potential for treating diabetes mellitus at its T2DM stage because oxidative stress alongside chronic inflammation and insulin resistance drive disease progression. Scientific research indicates that these compounds adjust insulin signalling processes and control genes affecting glucose metabolism and reduce inflammatory cytokine activities (Salau et al., 2021a) (Table 1).

**TABLE 1:** Effects of different compounds on gene expression related to diabetes mellitus.

Bioactive compounds	Biological activity	Mechanisms of action for Vanillin	References
Vanillin (150 and 300 mg/kg) for 5 weeks	Antioxidant, antidiabetic	↓ Glutathione, glycogen content, superoxide dismutase and catalase enzyme activities	Salau et al. (2021b)
Ferulic acid (200 mg/kg, intragastric, once daily) for 8 weeks	Anti-inflammatory, antidiabetic	↓ Blood glucose levels, inhibited expressions p62, NOD-like receptor family pyrin domain containing 3 (NLRP3) and interleukin-1β (IL-1β) in renal tissues	Ma et al. (2022)
Vanillin	Antioxidant, anti-inflammatory	Enhances insulin sensitivity, reduces Inflammation via ↓ NF-κB, ↑ AMPK activation, ↑ GLUT4 translocation	Oladimeji et al. (2022)
Ferulic acid (150 or 300 mg/kg) for 5 weeks	Antioxidant, anti-inflammatory	Significant (p < 0.05) decreased blood glucose level, with concomitant increase in serum insulin level. ↑ GSH, HDL-C, SOD, and catalase activities	Salau et al. (2022)
Tannins	Antioxidant, gut microbiota modulation	Enhances insulin sensitivity via gut–liver axis by binding proteins and enzymes, and modulates inflammation	Akpoveso et al. (2023)
Flavonoids	Antioxidant, anti-obesity, anti-inflammatory	Reduces insulin resistance, supports weight control by inhibiting lipid peroxidation, regulates adipogenesis	Akpoveso et al. (2023)
Vanillic acid	Antioxidant, anti-inflammatory, inhibits lipid peroxidation	Neutralizes ROS, protects pancreatic beta cells, inhibits lipid peroxidation by ↑ Nrf2 pathway, ↓ ROS, modulates PPARγ and PI3K/Akt pathways	Momtaz et al. (2023)
Vanillin (150 mg/kg) or 300 mg/kg) for 5 weeks	Antidiabetic	Suppressed blood glucose levels, serum cholesterol, triglyceride, low-density lipoprotein cholesterol (LDL-c), alanine transaminase (ALT), aspartate transaminase (AST), creatinine, urea, uric acid, improved pancreatic β-cell function and glucose tolerance	Salau et al. (2024)

The agricultural production of *Vanilla planifolia* depends heavily on human labor for pollination while demanding precise climate factors and detailed post-harvest methods (Montero et al., 2022). The plant requires frequent use of plant protection products (PPPs) including fungicides and insecticides as well as herbicides because it remains highly vulnerable to diseases together with pests and environmental factors. The protection of yield quantities remains essential but concerns about pesticide remains and potential health hazards led to regulatory checks and demands to develop sustainable farming methods. The increasing demand for vanilla creates an immediate need to ensure environmentally friendly farming techniques which protect both therapeutic quality and food safety standards in the finished product.

### 7.1 Bioactive compounds of *Vanilla planifolia*

The bioactive compound over others that makes *Vanilla planifolia* distinctive is vanillin, which creates vanilla’s signature taste and aroma. As a component of vanilla flavor, vanillin serves important sensory roles and exhibits antioxidant and anti-inflammatory abilities. The powerful antioxidant properties of vanillin neutralize reactive oxygen species (ROS) through its demonstrated ability to reduce damaging molecules responsible for body oxidative stress. Studies confirm that oxidative stress fuels both insulin resistance and metabolic abnormalities as they appear in diabetes mellitus (Oladimeji et al., 2022).

Also, vanillin is a scavenger of ROS, which helps protect them from being damaged and reduces insulin resistance, leading to fewer complications of diabetes (Salau et al., 2021a). Vanillin has also been found to inhibit the secretion of proinflammatory cytokines such as TNF-α and IL-6, ultimately decreasing inflammatory response in glucose-transmitting tissues. The anti-inflammatory action is even more important for diabetic patients due to its clinical effectiveness on general metabolic health (Singh et al., 2022).

### 7.2 *In vitro* study

*Vanilla planifolia* contains ferulic acid (also called 4-hydroxy-3-methoxycinnamic acid), another important bioactive compound. Ferulic acid is a phenolic acid found in the cell walls of plants, including vanilla beans, with antioxidants and anti-inflammatory properties (Xie et al., 2022). Ferulic acid also has impressive antioxidant qualities, similar to vanillin. It scavenges ROS and aids in the reduction of oxidative stress, one of the primary causes of the diabetic complications like neuropathy (Dhaliwal et al., 2020). As a powerful antioxidant, ferulic acid decreases oxidative damage and protects the cells of the body, including those of the pancreas that produce insulin (pancreatic beta cells). Ferulic acid improves glucose metabolism by enhancing insulin sensitivity. According to studies, ferulic acid may increase the action of insulin and improve glucose uptake by the cells, helping to maintain the balance of blood glucose. Therefore, ferulic acid can be a valuable compound in cases of T2DM in which insulin resistance plays a prominent role (Salau et al., 2022).

*Vanilla planifolia* has other polyphenols, such as tannins and flavonoids, that are also good for managing diabetes, aside from some of the earlier mentioned major compounds like vanillin and ferulic acid. Polyphenols trigger the body’s antioxidant defence system, promoting the activity of enzymes such as superoxide dismutase (SOD) and catalase that counteract oxidative damage. Therefore, this is valuable in diabetes, in which oxidative stress is a major pathological event in the pathogenesis of insulin resistance (Akpoveso et al., 2023). Polyphenols also inhibit lipid peroxidation, protecting cellular membranes as well as mitochondrial integrity. Such a function is critical in T2DM where lipid disorder and free fatty acid buildup lead to persistently increased insulin resistance (Naz et al., 2023). Tannins may enhance insulin sensitivity through gut–liver axis by binding proteins and enzymes, as well as modulate inflammation (Akpoveso et al., 2023).

Vanillic acid (4-hydroxy-3-methoxybenzoic acid) is a strong antioxidant and a vanillin metabolite that protects the organism from oxidative damage. Vanillic acid can effectively neutralize ROS, thus mitigating oxidative stress on tissues (Momtaz et al., 2023). This activity prevents oxidative damage in the pancreatic beta cells involved in insulin secretion. It also inhibits lipid peroxidation by increasing Nrf2 pathway, and modulating PPARγ and PI3K/Akt pathways (Table 1), thus prevents membrane damage and enhances cell function, which is particularly important in diabetes and its complications (Khairnar et al., 2021).

### 7.3 *In vivo* study

A study by Salau et al. (2021b) evaluated the therapeutic potential of vanillin in mitigating muscle dysmetabolism associated with T2DM in male Sprague–Dawley rats. The study showed that induction of T2DM in rats resulted in pronounced redox imbalance, evidenced by reduced glutathione content, diminished activities of antioxidant enzymes (superoxide dismutase (SOD) and catalase) and elevated malondialdehyde and nitric oxide levels. Oral administration of vanillin at low (150 mg/kg) and high (300 mg/kg) doses for 5 weeks significantly reversed biochemical and metabolic disturbances, restoring antioxidant defenses, normalizing enzyme activities, reactivating lipid metabolic pathways, and improving glycogen levels (Table 1).

Moreover, vanillin enhanced muscle glucose uptake *ex vivo*, with effects comparable to metformin. This finding suggests that vanillin mitigates oxidative stress, inflammation, cholinergic and purinergic dysfunctions, while modulating the glucose-lipid metabolic switch in diabetic rats. In another study, Salau et al. (2024) found that administration of 150 mg/kg and 300 mg/kg of vanillin for 5 weeks reduced blood glucose levels. Vanillin not only reduced blood glucose levels, but also increased serum and pancreatic tissue glutathione (GSH) levels, enhanced SOD and catalase activities, and elevated hepatic glycogen content, demonstrating its potential to improve antioxidant defense and metabolic regulation in diabetes.

Diabetic nephropathy, a major microvascular complication of diabetes mellitus. Ferulic acid (200 mg/kg, administered orally for 8 weeks) exerted significant nephroprotective effects in mice with high-fat diet/streptozotocin-induced diabetic nephropathy. Ferulic acid also improved lipid metabolism by decreasing triglycerides, total cholesterol, low-density lipoprotein cholesterol (LDL-C), and high-density lipoprotein cholesterol (HDL-C). At the molecular level, FA enhanced autophagy, as indicated by increased light chain 3 (LC3) expression, while suppressing p62 accumulation and downregulating inflammatory mediators including NOD-like receptor family pyrin domain containing 3 (NLRP3) and IL-1β in renal tissues. Li et al. (2020) demonstrated that inhibition of NLRP3 inflammasome activation attenuates renal inflammation and fibrosis in diabetic nephropathy mice. This finding indicates that ferulic acid may inhibit the renal inflammatory process in diabetic mice by downregulating IL-1β and NLRP3 protein expression, thereby exerting a kidney-protective effect (Ma et al., 2022).

A similar finding was also reported by Salau et al. (2022), in which oral administration of ferulic acid at 150 or 300 mg/kg for 5 weeks significantly reduced blood glucose levels while increasing serum insulin concentrations in male Sprague-Dawley rats using the fructose-streptozotocin model. Reduction in blood glucose levels, elevation in serum insulin concentrations, and attenuation of insulin resistance in ferulic acid–treated diabetic rats collectively suggest that this phenolic compound has the potential to improve glucose homeostasis in T2DM. This effect is further evidenced by enhanced glucose tolerance, amelioration of pancreatic β-cell dysfunction, and restoration of pancreatic histology in diabetic rats. These findings are consistent with previous reports, which demonstrating that ferulic acid reduces blood glucose levels, accompanied by the upregulation of insulin signaling molecules in diabetic models (Narasimhan et al., 2015).

The bioactive compounds of *Vanilla planifolia*, particularly vanillin, exhibit rapid gastrointestinal absorption but undergo extensive hepatic metabolism, primarily glucuronidation and sulfation, which results in low systemic bioavailability. Pharmacokinetic studies indicate fast clearance and urinary excretion of conjugated metabolites (Arya et al., 2021). These characteristics limit therapeutic potential, and further research is required to elucidate the formulation strategies to enhance bioavailability.

### 7.4 *Vanilla planifolia* and its therapeutic potential

**FIGURE 2:**
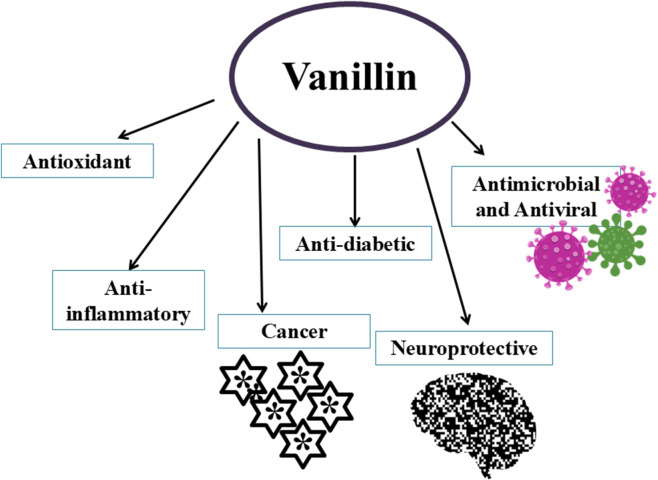
Potential health benefits of vanillin.

The antioxidant properties make *Vanilla planifolia* valuable to human health. The plant produces three abundant bioactive phenolic compounds, including vanillin along with ferulic acid and vanillic acid, which act as reactive oxygen species (ROS) scavengers. ROS function as highly reactive particles, yet their unchecked rise can cause cellular and tissue damage, which leads to multiple chronic conditions, including diabetes, cardiovascular diseases, and neurodegenerative disorders (Arya et al., 2021). Research shows that vanillin acts as an antioxidant in diabetes by protecting the insulin-producing beta cells in the pancreas. Research indicates that vanillin helps protect insulin secretion along with maintaining glucose homeostasis by stopping damage from oxidative processes (Dinić et al., 2022). The antioxidant effects of *Vanilla planifolia* are supported by ferulic acid as a dominant compound that blocks lipid peroxidation, which generates free radicals and damages cellular lipids. The compound ferulic acid protects against insulin resistance and provides enhanced glucose metabolic functions, particularly in liver and muscle tissues, through its ability to reduce lipid peroxidation (Zhang et al., 2022). Figure 2 shows the potential health benefits of vanillin.

The development toward insulin resistance which serves as the essential foundation for Type 2 diabetes mellitus relies heavily on persistent pro-inflammatory conditions. Cytokines TNF-α, IL-6 and IL-1β create disruption within the insulin pathway signal before triggering beta cell dysfunction (Reinehr, 2019). Effective insulin sensitivity and blood glucose management need the reduction of inflammatory responses. In *Vanilla planifolia*, the anti-inflammatory compounds vanillin and cinnamic acid suppress NF-κB pathway activity to achieve their anti-inflammatory function. The NF-κB pathway functions as a fundamental regulatory mechanism of inflammation. The pro-inflammatory cytokines TNF-α and IL-6 decrease through inhibition of this pathway according to Park et al. (2022). Ferulic acid demonstrates dual functions as an antioxidant and possesses anti-inflammatory properties. The reduction of pro-inflammatory genes by ferulic acid decreases the inflammatory burden affecting adipose tissue muscle and liver and promotes insulin resistance development (Xie et al., 2022).

The bioactive compounds located in *Vanilla planifolia* that consist of vanillin and ferulic acid and polyphenols demonstrate exceptional anti-diabetic characteristics that support both insulin sensitivity and glucose metabolic processes. Insulin resistance in T2DM is reduced by oxidative stress due to the antioxidant vanillin which deactivates reactive oxygen species (ROS). Studies found that this compound can improve glucose metabolism by increasing cellular insulin sensitivity and enhancing glucose transport which leads to better blood sugar regulation. Polyphenolic substances of *Vanilla planifolia* including flavonoids and tannins that activate the body’s antioxidant defense mechanisms to protect pancreatic beta cells and other cells from oxidative damage and inflammatory processes, which are essential diabetes complications risk factors. Evidence showed that *Vanilla planifolia* works synergistically to stop the formation of T2DM thus representing a beneficial natural therapy for managing blood glucose parameters and resisting insulin resistance (Oladimeji et al., 2022).

Oxidative damage, inflammation, and metabolic dysfunction are well known to link chronic diseases such as diabetes to neurodegenerative conditions including Alzheimer’s disease and Parkinson’s disease. *Vanilla planifolia* may reduce the risk of cognitive decline or improve brain health due to its neuroprotective properties. Vanillin and ferulic acid have neuroprotective effects by alleviating oxidative stress and neuroinflammation, both operating in the pathophysiology of neurodegenerative diseases. These compounds protect nerve cells from oxidative damage and improve cognitive function, which may be impaired in people with diabetes due to high glucose and metabolic dysfunction (Ghaderi et al., 2024). Particularly, ferulic acid has been identified to improve cognitive memory and reduce inflammation in neurodegeneration and thus can be used as a promising agent in preventing neurodegenerative diseases associated with chronic metabolic diseases (Mei et al., 2023).

*Vanilla planifolia* demonstrates both antimicrobial properties and antiviral actions. Study showed that vanillin inhibited both Gram-positive and Gram-negative bacterial pathogens, including *Staphylococcus aureus* and *Escherichia coli*, which commonly cause diabetic infections. The infection-management properties of vanillin stem from its ability to block bacterial growth that could worsen diabetic symptoms. The antiviral activities of this substance against diabetes-aggravating viruses are limited, leading to few benefits as an apple cider vinegar weight loss tool (Tawfeeq and Qassir, 2020).

Vanillin has shown the potential to prevent mutations and genotoxicity (Olatunde et al., 2022), which are crucial factors in cancer development. Vanillin effectively prevents mutations caused by harmful chemicals like methyl methane and mitomycin C (Olatunde et al., 2022). Experimental studies using mouse bone marrow cells and *Drosophila melanogaster* (fruit flies) revealed that vanillin reduces genotoxicity and prevents DNA recombination, a process that can lead to mutations (Olatunde et al., 2022). In addition, vanillin mitigated damage induced by mutagens such as N-ethyl-N-nitrosourea and bleomycin in fruit flies. The anticancer properties of vanillin have been observed in several studies. For instance, when tested on colorectal cancer cells (HT-29), vanillin arrested the cell cycle at the G2/M and G0/G1 stages and triggered apoptosis, signifying its potential in colorectal cancer prevention.

Similarly, studies on hamster lung cells indicated that vanillin reduced UV-induced DNA mutations and chromosomal damage, while enhancing DNA repair mechanisms. In human cancer cell lines like lung cancer (NCI-H460) and liver cancer (HepG2), vanillin suppressed metastasis and reduced gene expression related to tumor growth. Apart from that, as a potent antioxidant, vanillin neutralizes free radicals that can damage cells and DNA. Vanillin protected DNA from radiation-induced damage in both laboratory and animal models. The free radical scavenging ability underscores its importance in preventing oxidative stress, a precursor to various diseases, including cancer (Olatunde et al., 2022).

Vanilla has historically been utilized for medicinal purposes across diverse cultures (Bythrow, 2005). Recognizing its historical significance, numerous studies have investigated the therapeutic potential of *Vanilla* species and their main constituent, vanillin (Arya et al., 2021). Research has highlighted vanillin’s antibiotic properties and its potential role in treating sickle cell anemia (Abraham et al., 1991; Maisch et al., 2022). Notably, most studies have concentrated on vanillin’s anticarcinogenic properties, evidenced by its effects on neuroblastoma (SH-SY5Y), hepatocarcinoma (HepG2), and colorectal cancer (HT-29) cell lines, along with its antioxidant, cardioprotective, and antiviral properties (Tai et al., 2011; Hariono et al., 2016; Sirangelo et al., 2020).

A recent study showed that *Vanilla planifolia* extracts significantly decreased cell viability in HepG2 cells at dosage of 5,000 μg/mL for 72 h, 48 h, and 24 h. A similar effect was observed in mouse liver fibroblast (FC3H) cells (Araujo et al., 2024). In addition, ethanolic *Vanilla planifolia* leaf extract has also demonstrated antiproliferative activity against squamous carcinoma and breast cancer cells (Kaliappan and Kumaravelu, 2018; Vijaybabu and Punnagai, 2019). A study by Chang et al. (2024) evaluated the antitumor effect of *Vanilla planifolia* on glioblastoma multiforme cells. The study found that the ethanol extract of *Vanilla planifolia* stem significantly reduced the viability and colony-forming ability of glioblastoma multiforme cells *via* downregulation of 14 hub DEGs and upregulation of 9 hub DEGs.

### 7.5 Mechanisms of action

#### 7.5.1 Insulin signaling pathway

The insulin pathway is an important regulator of glucose uptake in cells and its subsequent homeostasis in the blood. Under normal conditions, insulin attaches to its corresponding receptor on the cell membrane, spurring a cascade of intracellular pathways that enable absorption of glucose, particularly in muscle, adipose, and liver tissue (Norton, 2022). However, in the case of diabetes mellitus, particularly T2DM, insulin resistance inhibits this signalling pathway, resulting in increased blood glucose levels.

Vanillin and ferulic acid are both known to positively modulate the activity of genes related to the insulin signalling cascade, namely, the insulin receptor (INSR) and the insulin receptor substrate (IRS) proteins. Referred to as compounds A and B, these compounds augment the phosphorylation of IRS-1 at tyrosine residues, followed by enhanced downstream signalling via PI3K/Akt. This triggering cascade results in the translocation of GLUT4 (glucose transporter type 4) to the cell membrane to transport glucose into the cell. Vanillin and ferulic acid exhibit positive effects on these metabolic processes and pathways, contributing to improved insulin sensitivity that is typically reduced in insulin-resistant cells in diabetic individuals (Zhang et al., 2021).

Ferulic acid is a strong antioxidant and anti-inflammatory agent that regulates various metabolic pathways associated with glucose homeostasis. The expression level of essential glucose-phosphorylating enzyme glucokinase (GK) increases in liver and pancreas because of this treatment. Measurements of glucokinase activity lead to higher glucose consumption and reduced hepatic glucose production. Through the AMPK pathway, ferulic acid elevates cellular glucose absorption and activates glycogen synthesis together with more effective glucose utilization in insulin-sensitive tissues (Khatun, 2024).

**FIGURE 3:**
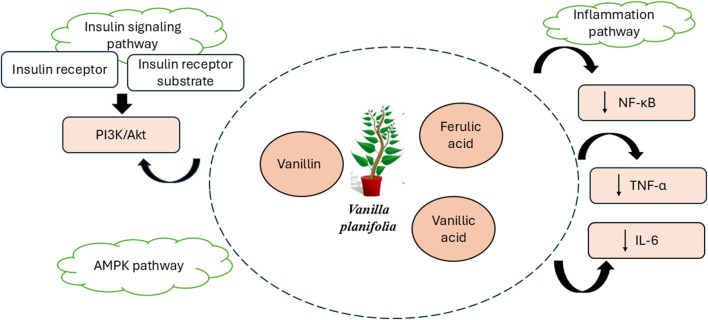
Molecular targets of *Vanilla planifolia* phytochemicals in diabetes.

Metabolic precursor vanillin produces its important biochemical impact on glucose metabolism through vanillic acid. Acting within insulin-sensitive tissues vanillic acid delivers protection from oxidative stress while simultaneously blocking liver-generated glucose. Vanillic acid reduces hepatic lipid accumulation by preventing the depletion of glycogen synthesis during hyperinsulinemia and activates glucokinase (GK) to reduce deficiencies in glucose metabolism in liver cells, one of the primary factors contributing to insulin resistance in T2DM (Sreelekshmi and Raghu, 2021). This leads to improved glycaemic control and enhanced insulin sensitivity due to an increase in vanillic acid. The molecular targets of *Vanilla planifolia* phytochemicals in diabetes is shown in Figure 3.

#### 7.5.2 Inflammatory pathways

T2DM is associated with a persistent inflammation which contributes to insulin resistance. Key pro-inflammatory cytokines such as TNF-α, IL-6 and IL-1β impair insulin signalling by promoting β cell dysfunction. Reducing inflammation is important for helping body to respond to insulin and regulating glucose (Reinehr, 2019).

Vanillin shows strong anti-inflammatory activity that correlates well with modulation of the nuclear factor kappa B (NF-κB) pathway, a major player in controlling inflammation. These compounds are known to repress the signal transduction of pro-inflammatory cytokines, including TNF-α and IL-6, prominent contributors to both insulin resistance and beta cell dysfunction in diabetes (Figure 3). Through the inhibition of NF-κB activation, vanillin and cinnamic acid reduce systemic inflammation, which leads to an improvement in insulin sensitivity and glucose metabolism (Gandhi et al., 2024).

**FIGURE 4:**
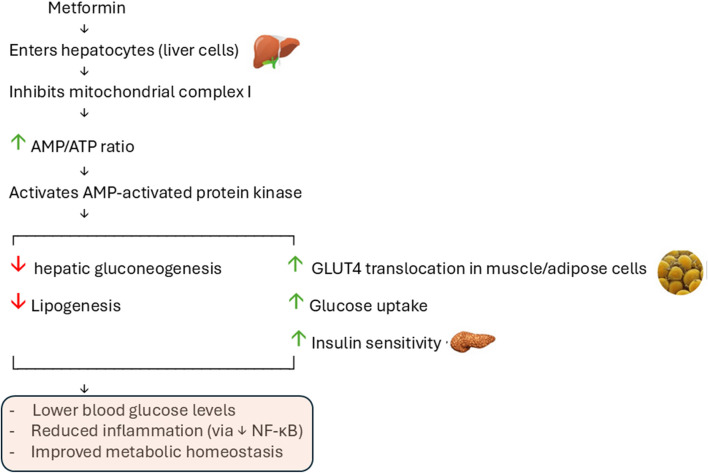
Proposed mechanism of action of metformin in combating diabetes. Metformin inhibits mitochondrial complex I, leading to AMPK activation. This reduces hepatic gluconeogenesis and lipogenesis, while enhancing GLUT4 translocation and glucose uptake in peripheral tissues. Therefore, it improved insulin sensitivity and reduced blood glucose levels.

Tannins alongside flavonoids represent two groups of polyphenolic compounds essential for managing inflammatory biological reactions. The polyphenols lower the activation of NF-κB signaling while blocking inflammatory cytokine secretion by adipocytes and glucose metabolism-related tissues. The consumption of polyphenolic compounds leads to better inflammatory gene expression (Olędzka and Czerwińska, 2023). Figure 4 shows the proposed mechanism of action of metformin in combating diabetes.

## 8 Safety and toxicity

The natural vanilla market, generating millions in annual revenue, is largely dependent on *Vanilla planifolia*, a species with limited genetic diversity and vulnerability to pathogens. With growing consumer demand for natural vanilla, recognized for its rich and authentic flavor profile, there is a critical need for alternative production methods to ensure a stable and sustainable supply. In this regard, wild relatives of vanilla offer a promising avenue for expanding and securing natural vanilla production. However, these wild vanilla species must be thoroughly assessed for toxicity to identify any potential risks and confirm their safety for consumption.

Vanilla extract, oil, seed powder, and vanillin are classified as Generally Recognized as Safe (GRAS) by the U.S. Food and Drug Administration (FDA) for use as spices, natural seasonings, and flavoring agents in food (section 409 of the Federal Food, Drug, and Cosmetic Act [21 CFR 182.10; 21 CFR 182.60; CFR582.10]). Vanillic acid is approved as a food flavoring agent by the Joint FAO/WHO Expert Committee on Food Additives under designation number 959 (Joint FAO/WHO Expert Committee on Food Additives, 2002). Rats fed dietary vanillin at concentrations of 50 and 20 g/kg diet for 2 and 1 year, respectively, showed no adverse effects on hematology, growth, or tissue pathology (Hagan et al., 1967). Likewise, Ho et al. (2011) administered vanillin orally and intragastrically to rats at concentrations of 300 and 150 mg/kg for 14 weeks without observing any toxic effects.

A study by de Oliveira et al. (2024) evaluated the non-carcinogenic and non-mutagenic properties of ethanolic extracts of *Vanilla planifolia*, *Vanilla cribbiana*, *Vanilla chamissonis*, and *Vanilla bahiana*. The study showed that *Vanilla cribbiana* exhibited mutagenic effects at high doses (5,000 μg/plate) in the TA98 strain under conditions without metabolic activation. The study further revealed the safety of these extracts at low dosage (de Oliveira et al., 2024). Intriguingly, the study by Araujo et al. (2024) demonstrated that *Vanilla planifolia* induced mutations at the highest concentrations (4,000 and 5,000 µg/plate) in the TA98 strain. The mutagenic effects observed at higher concentrations of *Vanilla planifolia* extract may be attributed to its phenolic content and other organic compounds with antioxidant potential. At elevated concentrations, these compounds may induce a saturation effect inherent to biological systems. It is important to note that such concentrations far exceed those relevant to typical therapeutic or dietary exposure. Therefore, although high-dose mutagenicity highlights the need for caution in extrapolating safety margins, the findings do not necessarily undermine the potential therapeutic applications of *Vanilla planifolia* at physiologically achievable doses, where its antioxidant properties may confer therapeutic benefits.

Antioxidants, which typically scavenge free radicals, can also exhibit pro-oxidant behavior under certain conditions, as reported in studies on other plant extracts with high antioxidant content (Rietjens et al., 2005; Araújo-Lima et al., 2020). Furthermore, the complex mixture of bioactive compounds in plant extracts enables simultaneous interaction with multiple cellular targets and sites, potentially leading to adverse effects in biological systems (Gupta and Birdi, 2017). However, the doses tested in this study are significantly higher than typical human exposure to vanilla in food applications, reinforcing its safety under normal dietary conditions. A study reported that rats fed vanillin at 1.25 g/kg diet for 42 days exhibited reduced growth and reduced liver and blood activities of glutathione-S-transferase and superoxide dismutase (SOD) compared to controls (El-Wahab and Moram, 2012). The underlying reasons for the differing response to vanillin observed in this study, compared to previous findings, remain unclear.

Vanillin, one of the most abundant compounds in *Vanilla planifolia*, has been linked to potential anticarcinogenic, antimutagenic, and other biological properties (Rakoczy et al., 2021; Wang et al., 2024). Therefore, it is vitally important to understand the safety of vanillin. At relatively high concentrations, vanillin has not been associated with significant side effects (Lirdprapamongkol et al., 2005). A study demonstrated that vanillin administered orally or subcutaneously at doses up to 300 mg/kg for 14 weeks did not induce significant toxicity in rats (Ho et al., 2011).

Synthetic vanillin is commonly utilized in the food industry. Nonetheless, studies have shown that synthetic vanillin and other artificial ingredients may trigger *in vivo* toxicity in mice (Sales et al., 2018), for instance, genotoxic effects. While natural products contain various bioactive compounds, their presence does not inherently ensure safety. Vanillin, a secondary metabolite of *Vanilla* spp., is of significant economic importance due to its distinctive flavor and fragrance, making it highly valuable in the food and perfumery industries. Species with higher concentrations of vanillin and other key aromatic compounds, for instance, vanillic acid and *p*-hydroxybenzaldehyde, have contributed to the long-standing popularity and extensive consumption of this genus, maintaining its critical role in the global food industry (da Silva Oliveira et al., 2022).

The extracts of *Vanilla planifolia* are recognized as safe by the U.S. Food and Drug Administration (FDA) (FDA, 1977). Human exposure to these extracts is typically low, as they are primarily utilized as natural flavoring agents and spices. For instance, most baking recipes incorporate approximately one teaspoon of vanilla extract, equivalent to around 4 g (Sachdev, 2022).

## 9 Limitation of the study

The review highlights several compounds such as vanillin, vanillic acid, and other phenolics, but the full phytochemical profiles of *Vanilla planifolia* is not comprehensively studied. There are several limitations in this study, in which most of the finding comes from animal models or cell culture studies. There are no large-scale randomized controlled trials to confirm efficacy in diabetic patients. The synergistic effects between compounds remain unclear. The concentration of phytochemicals in *Vanilla planifolia* vary depending on geographic origin, extraction methods, and cultivation, which may confound the results. In addition, assessment of dose-response relationship and diabetes has not been evaluated. It would be useful to have moderate and high vanillin, vanillic acid, and other phenolics compared in the same study. Based on the evidence, the synergistic role played by compounds-derived from *Vanilla planifolia* worth study in-depth in a large clinical trial.

## 10 Conclusion and future perspective

Oxidative stress is a major risk factor in the development of diabetes. Various factors, including aging, unhealthy diets, and obesity, contribute to an oxidative environment that disrupts insulin sensitivity by increasing insulin resistance or impairing glucose tolerance. Blood sugar levels have significant effects on managing blood glucose levels and preventing chronic issues, especially when it comes to their lifestyle. Traditionally recognized for its flavor and fragrance, *Vanilla planifolia* is rich in bioactive compounds, including vanillin, vanillic acid, polyphenols, and phenolic acids, all of which display antioxidant, anti-inflammatory, and hypoglycemic properties. These compounds are thought to alter essential mechanisms involved in diabetes pathophysiology via mitigation of oxidative stress, increased insulin sensitivity and attenuation of inflammatory signalling that mediate glucose and lipid homeostasis. Beyond its effects on insulin sensitivity, vanilla’s antioxidant and anti-inflammatory properties may contribute to reducing the risk of long-term complications of diabetes. While more clinical studies are needed, these findings highlight the potential of vanilla as a complementary dietary component in diabetes management.

The bioavailability of *Vanilla planifolia* derived phytochemicals in humans remains uncertain, and natural variability in active compound concentrations across different sources of *Vanilla planifolia* may affect consistency of therapeutic outcomes. Furthermore, the potential for side effects, particularly with long-term or high-dose use, has not been fully evaluated. Future studies should focus on conducting randomized controlled clinical trials to confirm the efficacy and safety of *Vanilla planifolia* derived phytochemicals in diabetic patients.

Mechanistic investigations are needed to clarify how *Vanilla planifolia* and its bioactive compounds influence insulin signaling, protect pancreatic β-cells, and potentially modulate gut microbiota. Rigorous clinical trials are needed to establish efficacy, optimal dosage, and long-term safety. Variability in bioactive compound concentrations, limited data on bioavailability, and the potential for side effects with chronic use also required further investigation. It would be valuable to explore the synergistic or antagonistic effects of vanilla compounds when combined with other dietary phytochemicals or conventional antidiabetic drugs. In addition, novel formulation and delivery systems such as encapsulation or nanoparticle carriers could be developed to improve bioavailability. Despite phytochemicals may not function as pharmacological agents, they hold great promise and may provide leads for future approach to combat diabetes mellitus. The potential as alternatives or adjuncts to conventional therapies may warrant further investigation via long-term clinical trials.
